# Globally increased ultraconserved noncoding RNA expression in pancreatic adenocarcinoma

**DOI:** 10.18632/oncotarget.10242

**Published:** 2016-06-23

**Authors:** Jinmai Jiang, Ana Clara P. Azevedo-Pouly, Roxana S. Redis, Eun Joo Lee, Yuriy Gusev, David Allard, Dhruvitkumar S. Sutaria, Mohamed Badawi, Ola A. Elgamal, Megan R. Lerner, Daniel J. Brackett, George A. Calin, Thomas D. Schmittgen

**Affiliations:** ^1^ College of Pharmacy, University of Florida, Gainesville, FL, USA; ^2^ College of Pharmacy, Ohio State University, Columbus, OH, USA; ^3^ University of Texas M.D. Anderson Cancer Center, Houston, TX, USA; ^4^ Lombardi Cancer Center, Georgetown University, Washington, DC, USA; ^5^ Cambridge University, Cambridge, UK; ^6^ Veterans Affairs Medical Center, Oklahoma City, OK, USA; ^7^ Department of Surgery, University of Oklahoma Health Science Center, Oklahoma City, OK, USA; ^8^ Present address: Department of Molecular Biology University of Texas Southwestern Medical Center, Dallas, TX, USA; ^9^ Present address: College of Pharmacy and Wonkwang Oriental Medicines Research Institute, Wonkwang University, Republic of Korea

**Keywords:** noncoding RNA, ultraconserved elements, EGR1, pancreas cancer

## Abstract

Transcribed ultraconserved regions (T-UCRs) are a class of non-coding RNAs with 100% sequence conservation among human, rat and mouse genomes. T-UCRs are differentially expressed in several cancers, however their expression in pancreatic adenocarcinoma (PDAC) has not been studied. We used a qPCR array to profile all 481 T-UCRs in pancreatic cancer specimens, pancreatic cancer cell lines, during experimental pancreatic desmoplasia and in the pancreases of *P48^Cre/wt^*; *Kras^LSL-G12D/wt^* mice. Fourteen, 57 and 29% of the detectable T-UCRs were differentially expressed in the cell lines, human tumors and transgenic mouse pancreases, respectively. The vast majority of the differentially expressed T-UCRs had increased expression in the cancer. T-UCRs were monitored using an in vitro model of the desmoplastic reaction. Twenty-five % of the expressed T-UCRs were increased in the HPDE cells cultured on PANC-1 cellular matrix. UC.190, UC.233 and UC.270 were increased in all three human data sets. siRNA knockdown of each of these three T-UCRs reduced the proliferation of MIA PaCa-2 cells up to 60%. The expression pattern among many T-UCRs in the human and mouse pancreases closely correlated with one another, suggesting that groups of T-UCRs are co-activated in PDAC. Successful knockout of the transcription factor EGR1 in PANC-1 cells caused a reduction in the expression of a subset of T-UCRs suggesting that EGR1 may control T-UCR expression in PDAC. We report a global increase in expression of T-UCRs in both human and mouse PDAC. Commonalties in their expression pattern suggest a similar mechanism of transcriptional upregulation for T-UCRs in PDAC.

## INTRODUCTION

Pancreatic cancer is the fourth leading cause of cancer-related death in the USA [1]. Invasive pancreatic ductal adenocarcinoma (PDAC) accounts for more than 85% of all pancreatic tumors. PDAC has a 5 year survival rate of 7% [1] due to early dissemination and the rapid and insidious nature of growth and is predicted to be the second most lethal cancer in the USA by 2030 [2].

Like most cancers, PDAC arises from genetic alterations that are compounded by epigenetic mechanisms such as histone modifications and DNA methylation [3]. Whole genomic sequence analysis of 24 PDACs revealed that the four most frequently mutated genes were KRAS, p53, CDKN2A (p16) and SMAD4 [4]. KRAS was mutated in greater than 90% of the cases [4]. These findings were confirmed in the whole genome sequencing of pairs of PDAC and adjacent benign tissue from 3 patients and RNAseq from 2 tumors [5]. Both studies [4, 5] focused primarily on coding genes and transcripts.

It is widely accepted that non-coding RNAs play an important role in normal development, physiological homeostasis and disease [6]. Knowledge stemming from differential microRNA (miRNA) expression studies of PDAC [7–9] has been used to develop potential diagnostic [10, 11] and therapeutic applications [12, 13]. miRNA represents one of many classes of non-coding RNA that may play a role in the development, progression or metastasis of PDAC. Conceivably, other non-coding RNAs such as large intergenic non-coding RNAs (lncRNAs) [14], small nucleolar RNAs (snoRNAs), PIWI-interacting RNAs (piRNAs) and the transcribed-ultraconserved regions (T-UCRs) may also contribute to PDAC tumorigenesis.

T-UCRs are a set of 481 sequences that are strictly conserved among orthologous regions of the human, rat and mouse genome [15]. Nearly all of these gene segments are conserved in the chicken and dog genomes, with an average of 95 and 99% identity, respectively. They are at least 200 bp in length and are located in intergenic regions or within introns or exons of coding genes. The precise function of the T-UCRs is largely unknown. The T-UCR UC.283+A was shown to decrease pri-miR-195 biogenesis by impairing microprocessor recognition and pri-miRNA cropping [16]. We previously reported that many (~90%) of the T-UCRs are expressed in human cells and that T-UCR expression varied in a set of human leukemias and colorectal cancer specimens from patients [17]. Furthermore, the location of the T-UCRs was found in cancer associated genomic regions [17]. It has also been reported that single nucleotide polymorphisms (SNPs) and genetic alterations in UCRs may be associated with higher risk for familial breast cancer and colorectal carcinoma [18–20]. T-UCRs are differentially expressed in leukemias, hepatocellular carcinoma, neuroblastoma, prostate cancer and Barrett's esophagus [21–25]. To our knowledge, the expression of T-UCRs in PDAC has not been reported.

We used a qPCR low density array to profile the 481 T-UCRs in PDAC patient specimens and cell lines and in the pancreas of genetically engineered mice. We found that a large number of T-UCRs were transcribed in pancreas tissue and that a majority of these increase with disease progression. Several T-UCRs possessed oncogenic properties related to proliferation. Moreover, many T-UCRs were expressed similarly across different tissues suggesting that they share a common means of transcriptional and/or post-transcriptional regulation.

## RESULTS

### Increased T-UCR expression in PDAC tissues, cell lines and during experimental desmoplasia

**Figure 1:**
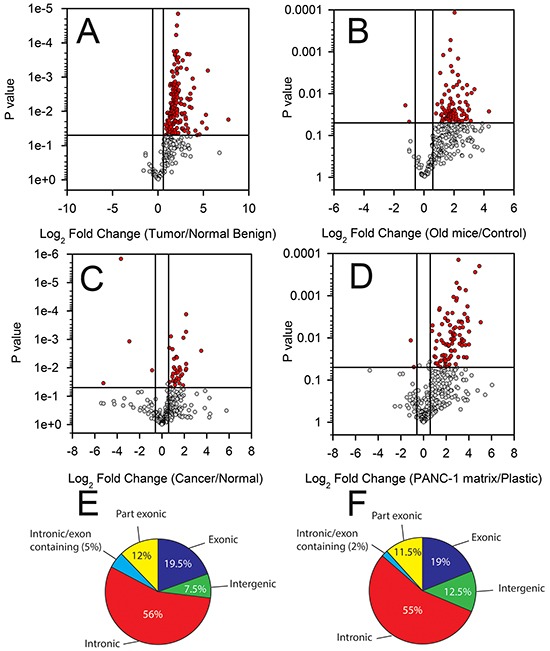
Differential T-UCR expression in PDAC tissues, cell lines and during experimental desmoplasia The T-UCR expression was determined by qPCR in **A.** normal, adjacent benign and PDAC tissues from patients, **B.** in the pancreas of control and P48^cre/wt^; Kras^LSL-G12D/wt^ (median 239 day old) transgenic mice, **C.** in PDAC and normal pancreas cell lines and **D.** during experimental desmoplasia. Expression values were converted to Log_2_ (fold change) and the Log_2_ (fold change) are compared to p-values using Volcano plots. Dots represent mean independent biological replicates for a given T-UCR. A threshold of P < 0.05 and fold change greater than 1.5-fold (red symbols) was applied to determine statistical significance. The genomic location of T-UCRs with increased expression in the PDAC compared to normal pancreas in human **E.** and in the pancreases of the old versus control transgenic mice **F.**

The expression of 481 T-UCRs was profiled in a discovery cohort of 20 pancreas specimens (4 normal, 8 adjacent benign and 8 PDAC). Of the 307 expressed T-UCRs in the human pancreas tissues, 175 (57%) were differentially expressed in the PDAC compared to normal and benign pancreas. All 175 of the differentially expressed T-UCRs were increased in the cancer and none were decreased (Figure 1A, Supplementary Table S1). The expression of many T-UCRs sequentially increased from normal to adjacent benign to PDAC. Representative examples are shown in Supplementary Figure S1A-S1C.

The T-UCR expression in old *P48^Cre/wt^*;Kras*^LSL-G12D/wt^* mice were compared to that of control mice. Similar to the PDAC patient specimens, a large proportion of the 328 detectable T-UCRs increased in the old *P48^Cre/wt^*;Kras*^LSL-G12D/wt^* versus control mice (95 T-UCRs were increased and 2 were decreased, Figure 1B, Supplementary Table S2). A trend of increasing T-UCR expression among the control, young and old *P48^Cre/wt^*;Kras*^LSL-G12D/wt^* mice was generally observed. T-UCR expression in the mice mosaic for Kras^G12D^ expression, *Pdx-1-Cre; Kras^LSL-G12D/wt^*, typically fell in between that of the old and young *p48^Cre/wt^*;Kras*^LSL-G12D/wt^* mice (Supplementary Figure S1D-S1F). This finding is not surprising due to the mosaic nature of Kras^G12D^ expression in the *Pdx-1-Cre; Kras^LS-LG12D/wt^* compared to *p48^Cre/wt^*;Kras*^LSL-G12D/+^* mice.

Comparing the T-UCR expression in the 4 PDAC cell lines to 2 normal pancreas cells showed a pattern of differential expression, though not as pronounced as in the tissues. Of the 307 detectable T-UCRs in the cell lines, 42 were differentially expressed (13.7%); 38 increased (90.5%) and 4 decreased (9.5%) (Figure 1C, Supplementary Table S3).

PDAC is frequently associated with an intense area of fibrosis known as the desmoplastic reaction composed of bands of fibrous stroma, collagen I and collagen II surrounding the malignant cells. The desmoplastic reaction may be studied experimentally by culturing cells on an extracellular matrix previously deposited by another cell [26]. To determine if the T-UCR expression is increased or decreased during desmoplasia, HPDE cells were cultured on a PANC-1 extracellular matrix. The T-UCR expression in HPDE cells cultured on the PANC-1 matrix was compared to that of HPDE cells cultured on plastic. Of the 383 expressed T-UCRs, 96 increased (25%) when HPDE cells were cultured on the PANC-1 matrix (> 1.5-fold, P < 0.05) while two (0.52%) had reduced expression (Figure 1D, Supplementary Table S4).

We next compared the genomic location of the T-UCRs that are increased in the mouse and human tissue data sets using the annotation as described by Mestdagh, et al., [27]. Specifically, we compared the T-UCRs that are increased in the PDAC compared to normal and benign tissue and in the pancreas of old versus control mice. Most of the increased T-UCRs are located in the intragenic regions (i.e. intronic, exonic and partly exonic) (Figure 1E, 1F). Over half of the increased T-UCRs in both comparisons were intronic T-UCRs while only 7 and 12% in the human and mouse data sets, respectively, were intergenic T-UCRs (Figure 1E, 1F). It is significant that such a large percentage of the T-UCRs with increased expression are intragenic as 39% of the 481 T-UCRs are classified as intergenic. In summary, the vast majority of the genes encoding T-UCRs that are increased in the mouse and human tissue data sets reside in intragenic rather than intergenic regions.

**Figure 2:**
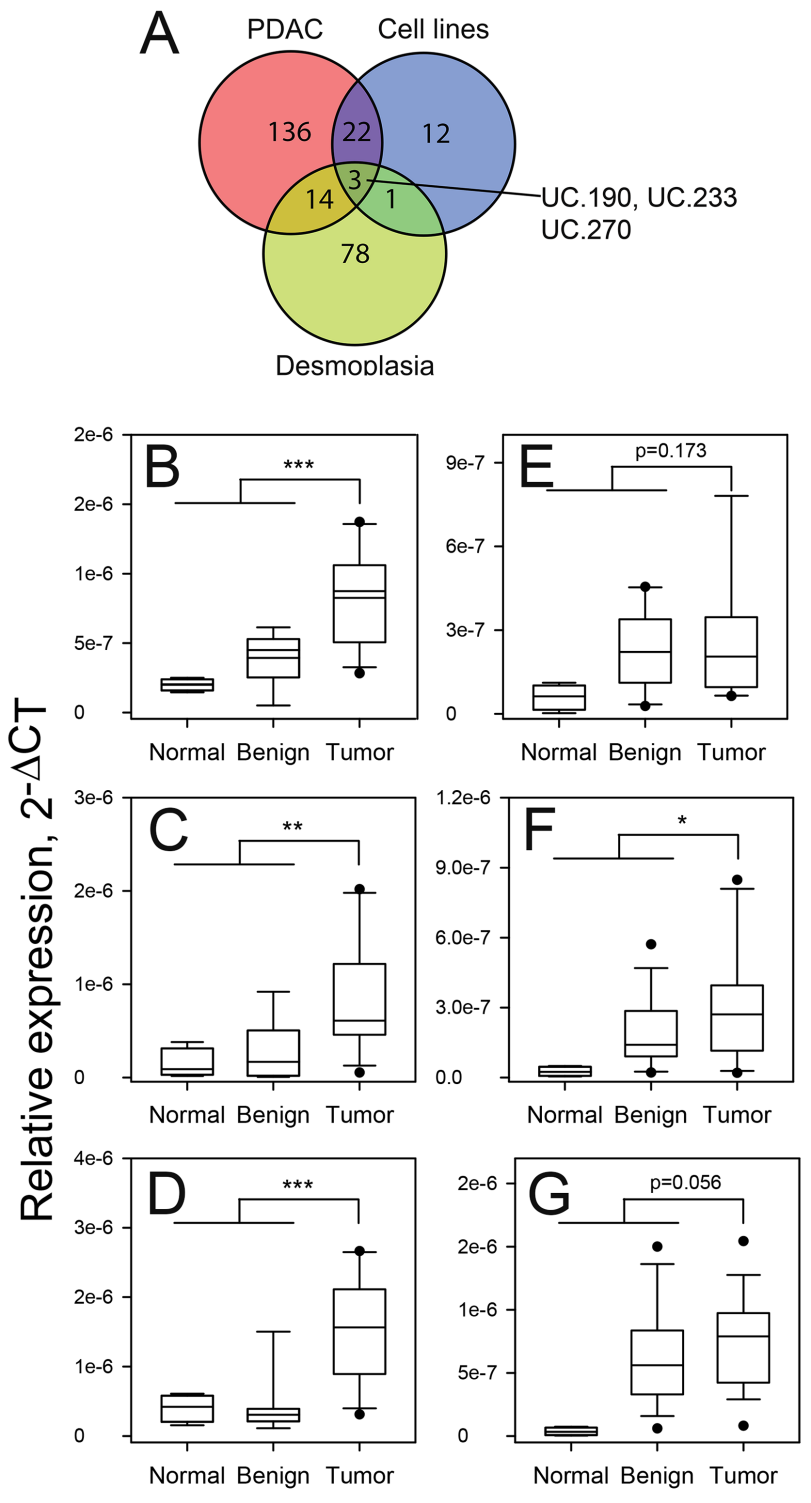
Expression of UC.190, UC.233 and UC.270 in discovery and validation cohorts of patients The T-UCR expression was determined by qPCR in PDAC tissue specimens, cell lines and during experimental desmoplasia. **A.** Overlap was determined in those T-UCRs with increased expression in each setting. Three T-UCRs (UC.190, UC.233 and UC.270) were increased in all three data sets. The expression of UC.190 **B, C.**, UC.233 **D, E.** and UC.270 **F, G.** in normal pancreas, adjacent benign tissue and PDAC was determined by qPCR in discovery (B, D, F) and validation (C, E, G) cohorts of patient tissues. * P < 0.05; ** P < 0.01; *** P < 0.001.

A comparison of all differentially expressed (increased) T-UCRs evaluated in the human cells/tissues showed that three T-UCRs (UC.190, UC.233 and UC.270) were increased in each of the data sets (Figure 2A). Shown in Figure 2 is the expression of UC.190, UC.233 and UC.270 in the PDAC tissues compared to normal and adjacent benign pancreas from the discovery cohort. We then determined the expression of these three T-UCRs in a validation cohort of 4 normal pancreas, 13 adjacent benign pancreas and 19 PDAC. The fold-change in expression of UC.190, UC.233 and UC.270 in the PDAC compared to the normal and benign pancreas was >1.5-fold. The p values for the UC.190, UC.233 and UC.270 comparisons (Tumor versus Normal and Benign) in the validation cohort were 0.173, < 0.05, 0.0562, respectively. The expression of UC.190, UC.233 and UC.270 in the validation cohort is presented in Figure 2. Comparing the UC.190, UC.233 and UC.270 expression in the control and old *P48^Cre/wt^*; Kras*^LSL-G12D/wt^* mice showed that the expression of all three T-UCRs were increased in the old *P48^Cre/wt^*; Kras*^LSL-G12D/wt^* versus control mice (Fold change > 1.5) however the p-value for UC.270 was not significant (Supplementary Figure S2).

### UC.190, UC.233 and UC.270 are required for sustained proliferation of pancreatic cancer cells

**Figure 3:**
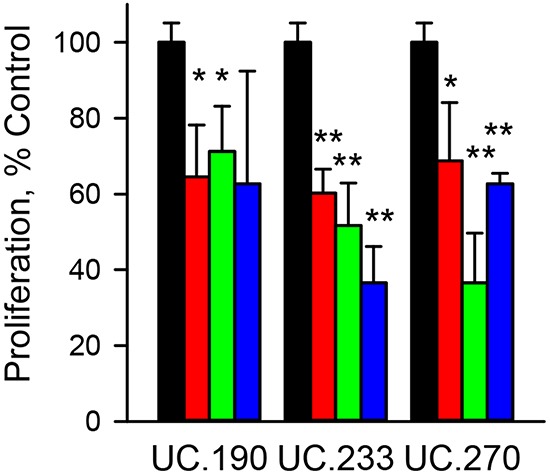
Effect of T-UCR knockdown on MIA PaCa-2 cells proliferation MIA PaCa-2 cells were exposed to 40 nM of control siRNA (black bars) or siRNA to UC.190, UC.233 or UC.270 for 96 h. Three different siRNAs - siRNA_1 (red), siRNA_2 (green) and siRNA_3 (blue) were evaluated for each T-UCR. The effect of the siRNA knockdown of T-UCRs on cell proliferation was compared to control siRNA. * P < 0.05; **P < 0.01. Representative data from duplicate experiments.

To explore the biological function of the T-UCRs, we next used the RNA knockdown approach. The three T-UCRs with increased expression (UC.190, UC.233 and UC.270, Figure 2) were selected for knockdown. The specific strand that UC.190, UC.233 and UC.270 are expressed from was determined by strand specific qPCR (data not shown). The results showed the following strand specific expression: UC.190 (plus strand), UC.233 (plus strand) and UC.270 (minus strand). For each T-UCR (UC.190, UC.233 and UC.270) we designed three distinct siRNAs targeting different expressed segments of the T-UCRs or one segment for the gapmers. The effect of the knockdown on T-UCR expression, T-UCR host gene expression and cell proliferation was evaluated. T-UCR expression was reduced by 60% or greater for all three siRNAs (Supplementary Figure S3A). T-UCR host gene expression was also reduced by the siRNA (Supplementary Figure S3B). Cell proliferation was measured following siRNA treatment at two different time points (72 h and 96 h) with the most significant reduction in viability occurring at 96 h. The mean reduction of all three siRNAs in MIA PaCa-2 cell proliferation following 96 h of siRNA exposure was 34%, 51% and 44% for the UC.190, UC.233 and UC.270, respectively (Figure 3). To further explore the effects of gene knockdown on cell proliferation we employed the gapmer oligo knockdown method. Gapmers to each T-UCR reduced cell proliferation from 30% to greater than 80% (Supplementary Figure S3C, S3E). Gapmers reduced the expression of each T-UCR up to 80% and reduced host gene expression up to 90% (Supplementary Figure S3D, S3F).

### Strong correlation of T-UCR expression in tissues

**Figure 4:**
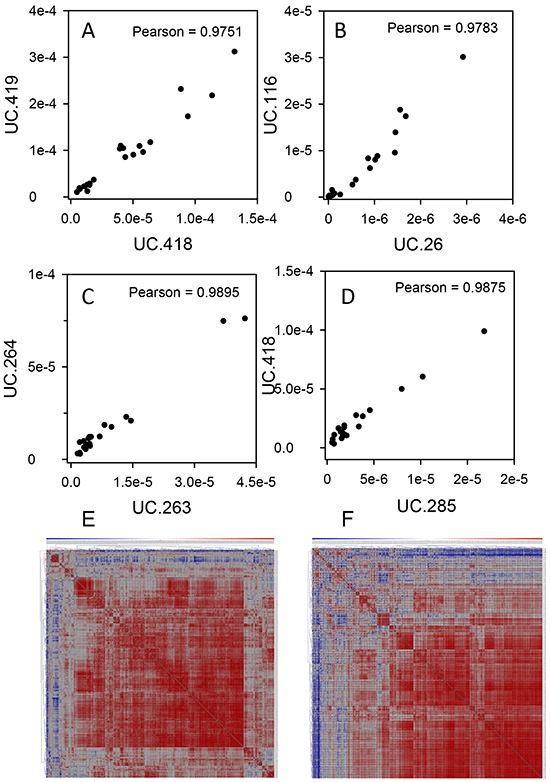
T-UCR have similar pattern of expression in both human and mouse tissues Correlations were determined for T-UCR expression in human **A, B.** and mouse **C, D.** tissues that are directly adjacent to one another (A, C) or on different chromosomes (B, D). The Pearson correlations were determined for all T-UCR gene expression in both human and mouse tissues. Of the 481 T-UCRs profiled by qPCR in 24 specimens of PDAC, adjacent benign and normal pancreas tissues, 307 were independently expressed. The pairwise Pearson correlation coefficients for all 307 expressed T-UCRs in these specimens were determined **E.** Of the 481 T-UCRs profiled by qPCR in pancreas of young and old *P48^Cre/wt^*; Kras*^LSL-G12D/wt^*, *Pdx-1-Cre; Kras^LSL-G12D/wt^* and control mice, 328 were independently expressed. The pairwise Pearson correlation coefficients for all 328 expressed T-UCRs in these specimens were determined **F.** Data are presented as heatmaps with red having the highest correlation and blue the lowest.

In neuroblastoma, specific T-UCRs displayed strong correlations in their expression [27], therefore, we wanted to look for similar trends of T-UCR expression in pancreatic cancer. The expression pattern among many T-UCRs in the mouse or patient tissues closely correlated with one another. This was to be expected for T-UCRs directly adjacent to one another in the genome that are likely transcribed as a unit such as human UC.418 and UC.419 (Figure 4A) or mouse UC.263 and UC.264 (Figure 4C). Interestingly, we found strong correlations in the expression of T-UCRs that are located on different chromosomes. For example, UC.26 and UC.116 (Figure 4B) in human and UC.285 and UC.418 (Figure 4D) in mouse. The pairwise Pearson correlation coefficients for all 307 and 328 expressed T-UCRs in the patient specimens and mouse pancreases, respectively, were determined and presented as heatmaps. The large portions of red color in each heatmap indicates a strong degree of expression correlation in both the human (Figure 4E) and mouse (Figure 4F) data. High resolution images of Figure 4E and 4F may be found as Supplementary Figure S4A and S4B, respectively.

### Transcriptional regulation of increased T-UCR expression

**Figure 5:**
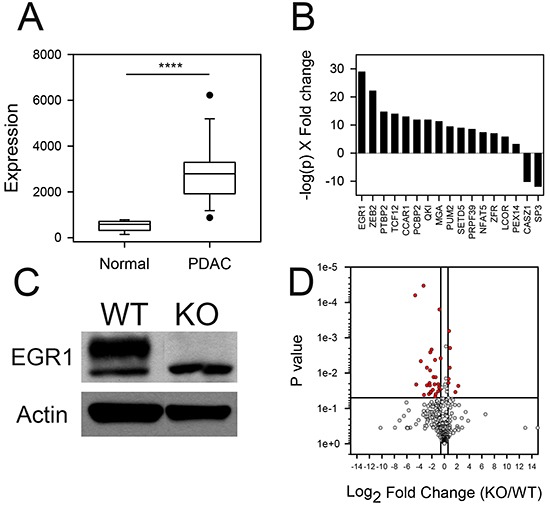
T-UCR expression in EGR1 knockout PANC-1 cells **A.** EGR1 expression in normal pancreas and PDAC from data set GSE71989. **B.** Differential expression of transcription factors in patient specimens of normal pancreas and PDAC (GSE71989 data set) ranked as -log(p)×FC. **C.** The CRISPR/Cas9 system was used to knockout the EGR1 gene in PANC-1 pancreatic cancer cells. The immunoblot of EGR1 and beta actin are shown in EGR1 knockout (KO) and wildtype (WT) PANC-1 cells. **D.** Expression values of T-UCRs in the EGR1 KO compared to WT cells were converted to Log_2_ (fold change) and the Log_2_ (fold change) are compared to p-values using a Volcano plot. Dots represent the mean of triplicate biological replicates for a given T-UCR. A threshold of P < 0.05 and fold change greater than 1.5-fold (red symbols) was applied to determine statistical significance, **** P < 0.0001.

The large increase in expression of many of the T-UCRs in pancreatic cancer suggests common means of regulation for these non-coding transcripts in the disease. Universal mechanism(s) for up-regulation for these T-UCRs would account for the global increase and correlation in expression between T-UCRs that reside in different chromosomes (Figure 4B, 4D). One possibility for the strong correlation among T-UCRs is that they are under control of similar transcription factors. To further investigate this, we identified transcription factors in human cancer cell lines by Encode RNA ChipSeq that bind to the putative promoter regions of the intronic and exonic T-UCR host genes. Up to 20 kbp of sequence upstream of the putative transcription start site for the intronic and exonic T-UCR host genes were examined. Intergenic T-UCRs were not evaluated since their promoters cannot be easily predicted. Of the 181 intronic and exonic T-UCRs examined, we identified several transcription factors that might be involved in transcribing these T-UCRs. To find the most informative transcriptional factors for functional studies, we compared the expression of these 17 putative transcription factors to our gene expression profiling data of human pancreas and PDAC (GSE71989). The expression of EGR1 (a transcriptional regulator that belongs to the family of C2H2-type zinc-finger proteins.) was highly increased in PDAC (Figure 5A) and had the lowest p-value and highest fold change in expression of the 17 transcription factors studied (Figure 5B, Supplementary Table S5). To functionally evaluate the role that EGR1 has on T-UCR expression in PDAC, EGR1 was successfully knocked out in the PANC-1 cells using the CRISPR/Cas 9 technology (Figure 5C). The expression of the 481 T-UCRs was profiled in both wild type and EGR1 knockout PANC-1 cells. The expression of 28 T-UCRs was reduced in the knockout cells (Fold change < 1.5, P < 0.05) compared to only 7 T-UCRs that were increased with EGR1 knockout (Figure 5D). These data suggests that EGR1 plays a role in regulating the transcription of a subset of the T-UCRs in pancreatic cancer.

## DISCUSSION

Differential expression of the T-UCR class of non-coding RNAs has been previously established in human cancer [17, 21–25]. We report that a large number of T-UCRs are differentially expressed in PDAC. The expression is predominately increased in human PDAC tissues and cell lines and in mouse models containing an activating Kras mutation. We identified three T-UCRs (UC.190, UC.233, UC.270) that were increased in all human models studied. siRNA or gapmer knockdown of these T-UCRs decreased cell viability, suggesting they might play a role in tumor promotion. T-UCRs themselves have been shown to have roles as gene enhancers in the mouse and bind miRNA in human cell lines [16, 17, 28]. It is conceivable, therefore, that T-UCRs play a regulatory role in promoting PDAC tumorigenesis by inducing oncogenes and/or down-regulating tumor suppressors.

The general pattern of T-UCR up-regulation in the different models of PDAC studied here is particularly striking. Of the many T-UCR expression profiling studies of human cancer to date [17, 21–23, 27, 29, 30], no similar trend of global T-UCR up regulation in solid tumors or leukemia/lymphoma exists. Of note, the expression of many T-UCRs, including those that reside on different chromosomes, correlated with one another in pancreatic tissues (Figure 4). This suggests a universal mechanism of T-UCR regulation in pancreatic cancer, perhaps through regulation by similar transcription factors. DNA hypermethylation resulted in T-UCR silencing in a variety of cancer cell lines and patient tumor specimens [31]. Since this study did not evaluate pancreatic cells or tissues, it is possible that the T-UCR upregulation may be unique to PDAC.

EGR1 was one transcription factor that we hypothesized could be responsible for regulating the T-UCR expression in PDAC (Figure 5). ChIP-Seq demonstrated that EGR1 globally increases miRNA expression in K562 cells stimulated with PMA [32]. EGR1 was increased in the patient data set (Figure 5A). EGR1 has been shown to be a stress response gene with increased expression in pancreatic acini following exposure to caerulein, an inducer of pancreatitis [33, 34]. The limitations of our experiment include transcription factor knockout in a cancer cell line and not in an organism. To definitely demonstrate EGR1 regulation of a subset of T-UCRs, the experiment would need to be repeated in an EGR1 knockout mice, preferably a pancreas specific conditional knockout. Since only a subset of T-UCRs were differentially expressed following EGR1 knockdown, other transcriptional or epigenetic regulation is likely. Mestdagh, et al., observed an association between H3K4me3 (a mark of active transcription) and the T-UCRs [27]. This type of epigenetic regulation is possible and may account for the global regulation of T-UCRS observed here. Also of note is that predominately intragenic rather than intergenic T-UCRs were increased in the mouse and human data sets (Figure 1E, 1F). This finding may offer clues as to the general regulation of intragenic and intergenic T-UCRs in PDAC.

Three T-UCRs were increased in the patient samples, during experimental desmoplasia, a mouse model of PDAC and pancreatic cancer cell lines (Figure 2A). All three increased UCRs, UC.190, UC.233 and UC.270, are intronic according to the Mestdagh, et al., annotation [27]. UC.190 is found within the FOXP4 gene, UC.233 within the AGAP3 gene and UC.270 is located in the MAPKAP1 gene. Examination of the gene expression profiling data (GSE71989) showed that the expression of these three T-UCR host genes did not increase in the PDAC compared to normal pancreas (data not shown). The genes encoding UC.190, UC.233 and UC.270 are on the same strand as the host genes. Since the expression of the host gene did not correlate with the T-UCR, it suggests that these three T-UCRs exists as transcripts that are separate from the T-UCR host gene pre-mRNA. Knockdown of the three T-UCRs using siRNA or gapmers reduced the proliferation of MIA PaCa-2 and PANC-1 cells as well as the host gene expression (Figure 3 and Supplementary Figure S3). Since both T-UCR and host gene mRNA were reduced by gene knockdown, we cannot rule out the possibility that knockdown of the host gene mRNA did not contribute to the reduction in cell proliferation.

We present here the first profiling study of T-UCRs in PDAC and identify a universal increase in this class of non-coding RNAs during disease progression in both human and mice. We further isolate three T-UCRs that are increased in multiple models of PDAC and lead to decreased cell viability when inhibited implicating UC.190, UC.233 and UC.270 as possible regulators of PDAC oncogenesis. These findings implicate T-UCRs as potential biomarkers or therapeutic targets for PDAC.

## MATERIALS AND METHODS

### Cells lines

The PDAC cells lines MIA PaCa-2, PANC-1, PL45 and SW1990 were obtained from the American Type Culture Collection (Manassas, VA). MIA PaCa-2, PL45 and PANC-1 cells were cultured using standard conditions with Dulbecco's Modified Eagle's Medium (DMEM) containing 10% fetal bovine serum (FBS) (Life Technologies, Grand Island, NY). SW1990 cells were cultured using Leibovitz's L-15 media supplemented with 10% FBS. The normal pancreatic ductal cell line HPDE was obtained from Dr. Ming-Sound Tsao, Ontario Cancer Institute, Toronto, Ontario. HPDE cells were cultured in keratinocyte medium containing 30 μg/ml of bovine pituitary extract and 0.2 ng/ml of human rEGF (Life Technologies, Grand Island NY). HPNE cells were a generous gift of Dr. Michel Ouellette, University of Nebraska and were cultured in Medium D as previously described [35]. All cells lines were successfully authenticated by Genetica DNA Laboratories, (Cincinnati, OH) or by the Interdisciplinary Center for Biotechnology Research, University of Florida (Gainesville, FL). Data are presented in Supplementary Table S6. Duplicate biological replicates of all cell lines were profiled.

### Tumor procurement

Pancreatic tissue samples were derived from patients undergoing a surgical procedure to remove a portion of the pancreas at the University of Oklahoma Health Sciences Center. Sample collection conformed to the policies and practices of the facility's Institutional Review Board. Sections from each specimen were examined by a pathologist to confirm that they were PDAC or unaffected benign tissue and were flash frozen in liquid nitrogen within 15 minutes of surgery. Samples denoted as normal pancreas were derived from subjects that expired from causes other than pancreatic disease and were purchased from various suppliers. Total RNA was extracted from the frozen tissues following pulverization in a cold mortar and pestle and total RNA was isolated using Trizol reagent (Invitrogen). The RNA integrity number (RIN) was determined using the Agilent 2100 Bioanalyzer. Patient demographics are listed in Supplementary Table S7.

### Transgenic mice

Transgenic mice harboring an activating Kras mutation were used. Total RNA from*Pdx-1-Cre;Kras^LSL-G12D/wt^*, *P48^Cre/wt^*; Kras*^LSL-G12D/wt^* and control mice was isolated using miRNeasy (Qiagen) according to the manufacturer's protocol and the RIN was determined using the Agilent Bioanalyzer. *Kras^G12D^* shows a consistent pancreatic expression under the P48 promoter, but shows a stochastic pattern of expression under the Pdx-1 promoter. In the *Pdx-1-Cre;Kras^LSL-G12D/wt^* mice low grade PanIN precursor lesions may arise as early as two weeks of age with PanIN-2 and PanIN-3 evident at 7-10 months [36]. The median age of the mice was 209 days (*Pdx-1-Cre;Kras^LSL-G12D/wt^*) and 34 (young) and 239 (old) days for *P48^Cre/wt^*; Kras*^LSL-G12D/wt^*. The median age of the control mice was 52 days. Detailed information on the mice may be found in Supplementary Table S8.

### qPCR

Total RNA was isolated from the cells or tissues using the Trizol protocol. RNA was treated with DNase I and cDNA was made from the RNA using SuperScript II reverse transcriptase (Invitrogen Carlsbad, CA) and random primers. Since we used random primers for the reverse transcription step, we are unable to determine the directionality of the transcription (i.e. plus or minus strand) from the profiling experiments. The expression of 481 T-UCRs was profiled in the cDNA using primers designed to each of the 481 T-UCRs. Primers were designed to hybridize to the sequence of the T-UCR genes as delineated by Berjano, et al., [15]. All primer sequences are included in Supplementary Table S9. qPCR was performed on an Applied Biosystems 7900 HT real-time PCR instrument that was equipped with a 384-well reaction plate using standard conditions. 18S rRNA or U6 snoRNA was used as the reference gene and data were analyzed using the comparative C_T_ method. To validate the normalizer for the qPCR, we report the C_T_ values for the various test and control groups for all experiments performed in the study. The data reported in Supplementary Figure S5 and Supplementary Table S10 demonstrates that the expression of the internal control gene was not significantly changed between the test and control groups (FC < 1.5, P > 0.05). A T-UCR was considered expressed in a particular sample if the mean C_T_ ≤ 36 and they were considered differentially expressed if the fold change between the comparative groups was greater than 1.5-fold and P < 0.05 (Student's t-test). To determine if the plus or minus strand was transcribed, strand specific qPCR was applied using sense or antisense RT primers as previously described [17].

### Gene expression profiling

The gene expression of 14 PDAC and 8 normal pancreas specimen RNAs was profiled on Affymetrix HG-U133 Plus 2 microarrays at the gene expression core lab at the Ohio State University Comprehensive Cancer Center. Gene expression raw data from Affymetric microarrays were processed and normalized using ‘affy’ R package from Bioconductor [37]. Data were normalized using the RMA algorithm [38], t-test was performed using R's built-in default statistical package. False discovery rate was controlled by utilizing q statistics using ‘qvalue’ R package from bioconductor. Gene expression data are presented in the GEO data set using the identifier GSE71989.

### Matrix deposit assay

Matrix deposit procedures were adapted from Ottaviano, et al [26]. HPDE cells were cultured on a matrix deposited by PANC-1 cell lines. To deposit the matrix, cell lines were plated at 10^5^ cells/well and cultured in 6-well plates for 72 hours. Cells were removed by treatment with 20 mM ammonium hydroxide for 5 minutes. After incubation, the wells were washed 3 times with sterile PBS. Removal of the cells was visually confirmed by microscopy. HPDE cells were cultured overnight in serum free medium and plated at 2x10^5^ cells/well in supplement free media over the PANC-1 matrix or plastic control. HPDE cells were incubated between 24 hrs prior to isolation of RNA.

### siRNA knockdown of T-UCRs

Transfections were performed in reverse with DharmaFECT 4 (Dharmacon). Briefly, DharmaFECT 4 was mixed with Opti-MEM and added to the 24 well-plate containing the siRNAs. The plate was incubated for 30 min on an orbital shaker to form a complex. Afterwards, 4,000 cells were added to each well. The final concentration of the siRNAs was 40 nM. At 72 h and 96 h, cells were washed with PBS, lysed and counted with the Beckman Coulter Cell Counter. RNA was collected 72 h after transfection and assayed by qPCR using U6 snoRNA as the normalizer. Viability after transfection was measured by using the WST-1 assay according to the manufacturer's protocol. All siRNA sequences used are included in Supplementary Table S11.

### Gapmer knockdown of T-UCRs

PANC-1 and MIA PaCa-2 cells were plated in 6 wells at a seeding density of 2 x 105 cells per well and left overnight to attach. The T-UCR gapmers were designed for individual T-UCR and ordered from Exiqon. Negative control A was used as the control for this study. The sequences for the 4 gapmers used are: Negative control A: 5′-AACACGTCTATACGC, UC.270: 5′-CCGCAGAGATAGAATT-3′, UC.233: 5′-GA GCGTGGAAAGCTAT-3′, UC.190: 5′-GTTAATTGCGG CCTTT-3′. T-UCR Gapmers were transfected at a concentration of 100 nM using Lipofectamine 3000. Forty-eight h post-transfection, cells were briefly washed with PBS, lysed using Trizol and assayed by qRT-PCR using gene specific primer as described above. For the proliferation assay, PANC-1 and MIA-PaCa-2 cells were plated at 2,500 cells per well in 96 well plates. The transfection concentration and procedure of transfection of the gapmers was similar to the knockdown experiment (100 nM). Cells were transfected and incubated for 96 h. Viability was measured using a WST1 assay.

### EGR1 knockdown in PANC-1 cells

EGR1 sgRNA cloning was carried out using the CRISPR/Cas9 procedure as described [39]. Guide RNAs were designed using the online CRISPR design tool (http://crispr.mit.edu/). Forward primer 5′- CACCGCTGCAGATCTCTGACCCGTT-3′ and Reverse primer 5′- AAACAACGGGTCAGAGATCTGCAGC-3′ were cloned into the bicistronic expression vector px459 which expresses both Cas9 mRNA and the sgRNA. PANC-1 cells were plated in a 6 well plate at a seeding density of 3 x 10^5^ cells/well. Lipofectamine LTX was used to transfect the vector containing both Cas9 and the sgRNA. Forty-eight hours post transfection, the cells were re-plated into another 6 well plate (1:5 dilution) and puromycin was introduced at a concentration of 1 μg/ml to the media to stably select the cells with EGR1 sgRNA expression. Stable colonies obtained after 7 days of puromycin selection were collected and screened for EGR1 protein expression using western blotting technique. EGR1 and beta actin antibodies numbers 4153 and 4970S, respectively, were used (Cell Signaling Technology, Danvers, MA).

### Data analysis

Differential expression analysis was performed using unpaired Student's t-test [40] with p-value threshold at 0.05 and additional criteria of fold change of at least 1.5. For both types of samples, human pancreatic tissue samples as well as mouse pancreatic tissues the pairwise Pearson correlation coefficients were calculated for each pair of expressed T-UCRs in these specimens. Using all pairwise correlation coefficients the correlation matrix was calculated and used to generate heatmaps. Heatmaps were generated using Multi-Array Viewer software package [41] with tri-color gradient scheme where gradient of red represents positive correlation, white color represent zero correlation and blue gradient represents negative correlation. Unsupervised hieratical clustering [42] was performed on both of these correlation matrixes (human and mouse) to determine clusters of T-UCRs that have most similar pattern of correlation across the samples.

## SUPPLEMENTARY MATERIALS FIGURES AND TABLES





## References

[1] Siegel RL, Miller KD, Jemal A (2015). Cancer statistics, 2015. CA Cancer J Clin.

[2] Rahib L, Smith BD, Aizenberg R, Rosenzweig AB, Fleshman JM, Matrisian LM (2014). Projecting cancer incidence and deaths to 2030: the unexpected burden of thyroid, liver, and pancreas cancers in the United States. Cancer Res.

[3] Omura N, Goggins M (2009). Epigenetics and epigenetic alterations in pancreatic cancer. Int J Clin Exp Pathol.

[4] Jones S, Zhang X, Parsons DW, Lin JC, Leary RJ, Angenendt P, Mankoo P, Carter H, Kamiyama H, Jimeno A, Hong SM, Fu B, Lin MT, Calhoun ES, Kamiyama M, Walter K (2008). Core signaling pathways in human pancreatic cancers revealed by global genomic analyses. Science.

[5] Liang WS, Craig DW, Carpten J, Borad MJ, Demeure MJ, Weiss GJ, Izatt T, Sinari S, Christoforides A, Aldrich J, Kurdoglu A, Barrett M, Phillips L, Benson H, Tembe W, Braggio E (2012). Genome-wide characterization of pancreatic adenocarcinoma patients using next generation sequencing. PLoS One.

[6] Esteller M (2011). Non-coding RNAs in human disease. Nat Rev Genet.

[7] Bloomston M, Frankel WL, Petrocca F, Volinia S, Alder H, Hagan JP, Liu CG, Bhatt D, Taccioli C, Croce CM (2007). MicroRNA expression patterns to differentiate pancreatic adenocarcinoma from normal pancreas and chronic pancreatitis. JAMA.

[8] Lee EJ, Gusev Y, Jiang J, Nuovo GJ, Lerner MR, Frankel WL, Morgan DL, Postier RG, Brackett DJ, Schmittgen TD (2007). Expression profiling identifies microRNA signature in pancreatic cancer. Int J Cancer.

[9] Szafranska AE, Davison TS, John J, Cannon T, Sipos B, Maghnouj A, Labourier E, Hahn SA (2007). MicroRNA expression alterations are linked to tumorigenesis and non-neoplastic processes in pancreatic ductal adenocarcinoma. Oncogene.

[10] Matthaei H, Wylie D, Lloyd MB, Dal Molin M, Kemppainen J, Mayo SC, Wolfgang CL, Schulick RD, Langfield L, Andruss BF, Adai AT, Hruban RH, Szafranska-Schwarzbach AE, Maitra A (2012). miRNA biomarkers in cyst fluid augment the diagnosis and management of pancreatic cysts. Clin Cancer Res.

[11] Schultz NA, Dehlendorff C, Jensen BV, Bjerregaard JK, Nielsen KR, Bojesen SE, Calatayud D, Nielsen SE, Yilmaz M, Hollander NH, Andersen KK, Johansen JS (2014). MicroRNA biomarkers in whole blood for detection of pancreatic cancer. JAMA.

[12] Park JK, Lee EJ, Esau C, Schmittgen TD (2009). Antisense inhibition of microRNA-21 or -221 arrests cell cycle, induces apoptosis, and sensitizes the effects of gemcitabine in pancreatic adenocarcinoma. Pancreas.

[13] Soubani O, Ali AS, Logna F, Ali S, Philip PA, Sarkar FH (2012). Re-expression of miR-200 by novel approaches regulates the expression of PTEN and MT1-MMP in pancreatic cancer. Carcinogenesis.

[14] Spizzo R, Almeida MI, Colombatti A, Calin GA (2012). Long non-coding RNAs and cancer: a new frontier of translational research?. Oncogene.

[15] Bejerano G, Pheasant M, Makunin I, Stephen S, Kent WJ, Mattick JS, Haussler D (2004). Ultraconserved elements in the human genome. Science.

[16] Liz J, Portela A, Soler M, Gomez A, Ling H, Michlewski G, Calin GA, Guil S, Esteller M (2014). Regulation of pri-miRNA processing by a long noncoding RNA transcribed from an ultraconserved region. Mol Cell.

[17] Calin GA, Liu CG, Ferracin M, Hyslop T, Spizzo R, Sevignani C, Fabbri M, Cimmino A, Lee EJ, Wojcik SE, Shimizu M, Tili E, Rossi S, Taccioli C, Pichiorri F, Liu X (2007). Ultraconserved regions encoding ncRNAs are altered in human leukemias and carcinomas. Cancer Cell.

[18] Yang R, Frank B, Hemminki K, Bartram CR, Wappenschmidt B, Sutter C, Kiechle M, Bugert P, Schmutzler RK, Arnold N, Weber BH, Niederacher D, Meindl A, Burwinkel B (2008). SNPs in ultraconserved elements and familial breast cancer risk. Carcinogenesis.

[19] Lin M, Eng C, Hawk ET, Huang M, Greisinger AJ, Gu J, Ellis LM, Wu X, Lin J (2012). Genetic variants within ultraconserved elements and susceptibility to right- and left-sided colorectal adenocarcinoma. Carcinogenesis.

[20] Lin M, Eng C, Hawk ET, Huang M, Lin J, Gu J, Ellis LM, Wu X (2012). Identification of polymorphisms in ultraconserved elements associated with clinical outcomes in locally advanced colorectal adenocarcinoma. Cancer.

[21] Braconi C, Valeri N, Kogure T, Gasparini P, Huang N, Nuovo GJ, Terracciano L, Croce CM, Patel T (2011). Expression and functional role of a transcribed noncoding RNA with an ultraconserved element in hepatocellular carcinoma. Proc Natl Acad Sci U S A.

[22] Fassan M, Dall'Olmo L, Galasso M, Braconi C, Pizzi M, Realdon S, Volinia S, Valeri N, Gasparini P, Baffa R, Souza RF, Vicentini C, D'Angelo E, Bornschein J, Nuovo GJ, Zaninotto G (2014). Transcribed ultraconserved noncoding RNAs (T-UCR) are involved in Barrett's esophagus carcinogenesis. Oncotarget.

[23] Hudson RS, Yi M, Volfovsky N, Prueitt RL, Esposito D, Volinia S, Liu CG, Schetter AJ, Van Roosbroeck K, Stephens RM, Calin GA, Croce CM, Ambs S (2013). Transcription signatures encoded by ultraconserved genomic regions in human prostate cancer. Mol Cancer.

[24] Sana J, Hankeova S, Svoboda M, Kiss I, Vyzula R, Slaby O (2012). Expression levels of transcribed ultraconserved regions uc.73 and uc.388 are altered in colorectal cancer. Oncology.

[25] Wojcik SE, Rossi S, Shimizu M, Nicoloso MS, Cimmino A, Alder H, Herlea V, Rassenti LZ, Rai KR, Kipps TJ, Keating MJ, Croce CM, Calin GA (2010). Non-codingRNA sequence variations in human chronic lymphocytic leukemia and colorectal cancer. Carcinogenesis.

[26] Ottaviano AJ, Sun L, Ananthanarayanan V, Munshi HG (2006). Extracellular matrix-mediated membrane-type 1 matrix metalloproteinase expression in pancreatic ductal cells is regulated by transforming growth factor-beta1. Cancer Res.

[27] Mestdagh P, Fredlund E, Pattyn F, Rihani A, Van Maerken T, Vermeulen J, Kumps C, Menten B, De Preter K, Schramm A, Schulte J, Noguera R, Schleiermacher G, Janoueix-Lerosey I, Laureys G, Powel R (2010). An integrative genomics screen uncovers ncRNA T-UCR functions in neuroblastoma tumours. Oncogene.

[28] Pennacchio LA, Ahituv N, Moses AM, Prabhakar S, Nobrega MA, Shoukry M, Minovitsky S, Dubchak I, Holt A, Lewis KD, Plajzer-Frick I, Akiyama J, De Val S, Afzal V, Black BL, Couronne O (2006). In vivo enhancer analysis of human conserved non-coding sequences. Nature.

[29] Ferdin J, Nishida N, Wu X, Nicoloso MS, Shah MY, Devlin C, Ling H, Shimizu M, Kumar K, Cortez MA, Ferracin M, Bi Y, Yang D, Czerniak B, Zhang W, Schmittgen TD (2013). HINCUTs in cancer: hypoxia-induced noncoding ultraconserved transcripts. Cell Death Differ.

[30] Watters KM, Bryan K, Foley NH, Meehan M, Stallings RL (2013). Expressional alterations in functional ultra-conserved non-coding RNAs in response to all-trans retinoic acid–induced differentiation in neuroblastoma cells. BMC cancer.

[31] Lujambio A, Portela A, Liz J, Melo SA, Rossi S, Spizzo R, Croce CM, Calin GA, Esteller M (2010). CpG island hypermethylation-associated silencing of non-coding RNAs transcribed from ultraconserved regions in human cancer. Oncogene.

[32] Wang W, Zhou D, Shi X, Tang C, Xie X, Tu J, Ge Q, Lu Z (2010). Global Egr1-miRNAs binding analysis in PMA-induced K562 cells using ChIP-Seq. Journal of biomedicine & biotechnology.

[33] Kaufmann A, Rossler OG, Thiel G (2014). Expression of the transcription factor Egr-1 in pancreatic acinar cells following stimulation of cholecystokinin or Galphaq-coupled designer receptors. Cellular physiology and biochemistry : international journal of experimental cellular physiology, biochemistry, and pharmacology.

[34] Kowalik AS, Johnson CL, Chadi SA, Weston JY, Fazio EN, Pin CL (2007). Mice lacking the transcription factor Mist1 exhibit an altered stress response and increased sensitivity to caerulein-induced pancreatitis. Am J Physiol Gastrointest Liver Physiol.

[35] Lee KM, Nguyen C, Ulrich AB, Pour PM, Ouellette MM (2003). Immortalization with telomerase of the Nestin-positive cells of the human pancreas. Biochem Biophys Res Commun.

[36] Hingorani SR, Petricoin EF, Maitra A, Rajapakse V, King C, Jacobetz MA, Ross S, Conrads TP, Veenstra TD, Hitt BA, Kawaguchi Y, Johann D, Liotta LA, Crawford HC, Putt ME, Jacks T (2003). Preinvasive and invasive ductal pancreatic cancer and its early detection in the mouse. Cancer Cell.

[37] Gautier L, Cope L, Bolstad BM, Irizarry RA (2004). affy–analysis of Affymetrix GeneChip data at the probe level. Bioinformatics.

[38] Irizarry RA, Hobbs B, Collin F, Beazer-Barclay YD, Antonellis KJ, Scherf U, Speed TP (2003). Exploration, normalization, and summaries of high density oligonucleotide array probe level data. Biostatistics.

[39] Wang H, Yang H, Shivalila CS, Dawlaty MM, Cheng AW, Zhang F, Jaenisch R (2013). One-step generation of mice carrying mutations in multiple genes by CRISPR/Cas-mediated genome engineering. Cell.

[40] Pan W (2002). A comparative review of statistical methods for discovering differentially expressed genes in replicated microarray experiments. Bioinformatics.

[41] Saeed AI, Sharov V, White J, Li J, Liang W, Bhagabati N, Braisted J, Klapa M, Currier T, Thiagarajan M, Sturn A, Snuffin M, Rezantsev A, Popov D, Ryltsov A, Kostukovich E (2003). TM4: a free, open-source system for microarray data management and analysis. Biotechniques.

[42] Eisen MB, Spellman PT, Brown PO, Botstein D (1998). Cluster analysis and display of genome-wide expression patterns. Proc Natl Acad Sci U S A.

