# Supplementary material for: Globally increased ultraconserved noncoding RNA expression in pancreatic adenocarcinoma
Source: Oncotarget. 2016 Jun 23;7(33):53165–77. doi: 10.18632/oncotarget.10242 (PMC5288176; doi:10.18632/oncotarget.10242)
Supplement: Supplementary file 1 [file oncotarget-07-53165-s001.pdf]

Globally increased ultraconserved noncoding RNA expression in pancreatic adenocarcinoma

SUPPLEMENTARY FIGURES AND TABLES

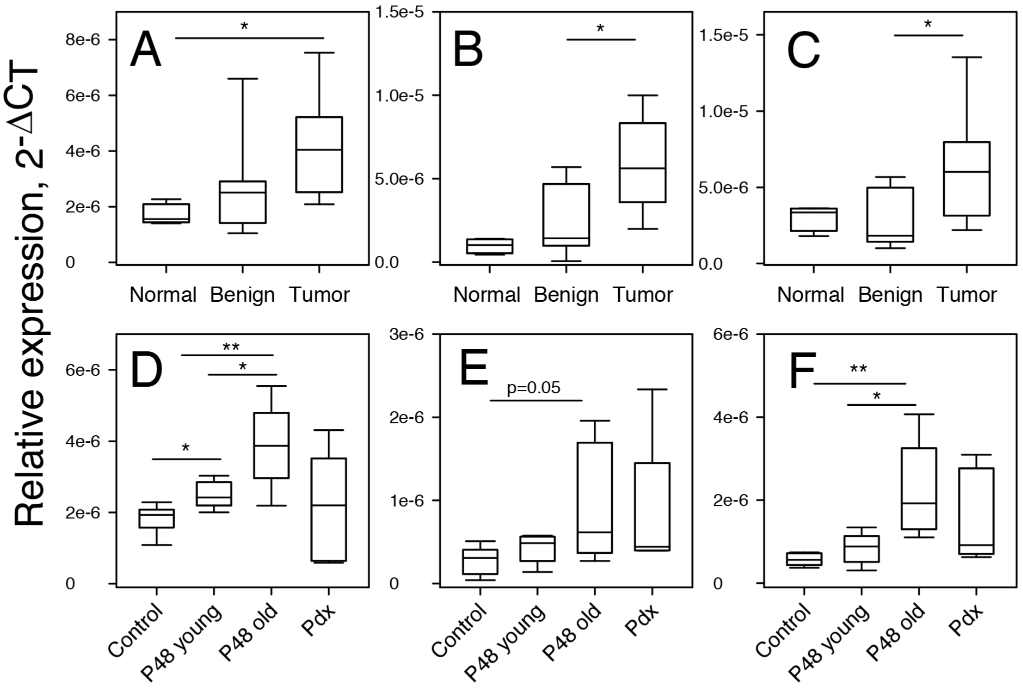

Supplementary Figure S1: T-UCR expression in PDAC and KRAS G12D tissues. The T-UCR expression was determined by qPCR in normal, adjacent benign and PDAC tissue from patients **A–C.** and in the pancreas of control, P48 young, P48 old and Pdx transgenic mice **D–F.** The relative expression of UC.74 (A, D), UC.104 (B, E) and UC.287 (C, F) are shown. * $P < 0.05$; ** $P < 0.01$.

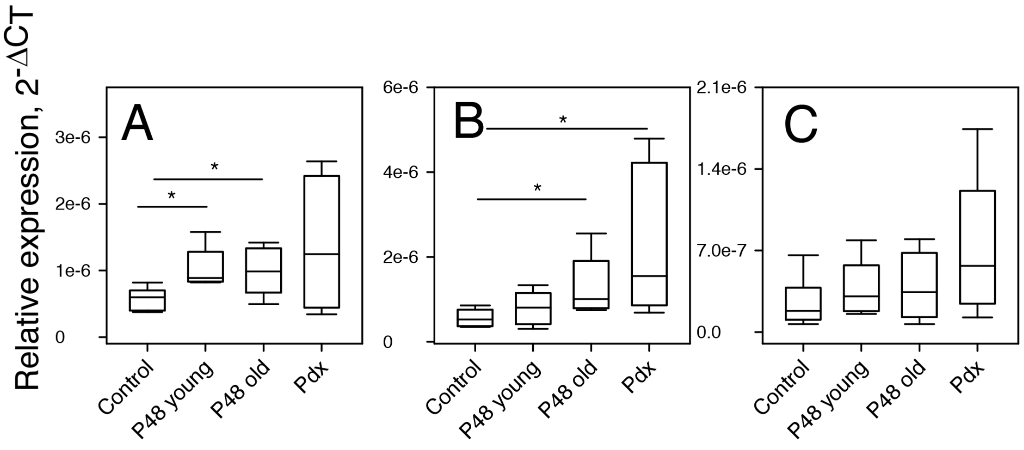

Supplementary Figure S2: Expression of UC.190, UC.233 and UC.270 in KRAS G12D tissues. The expression of UC.190 **A.** UC.233 **B.** and UC.270 **C.** was determined by qPCR in RNA isolated from the pancreas of control, P48 young, P48 old and Pdx transgenic mice. * $P < 0.05$.

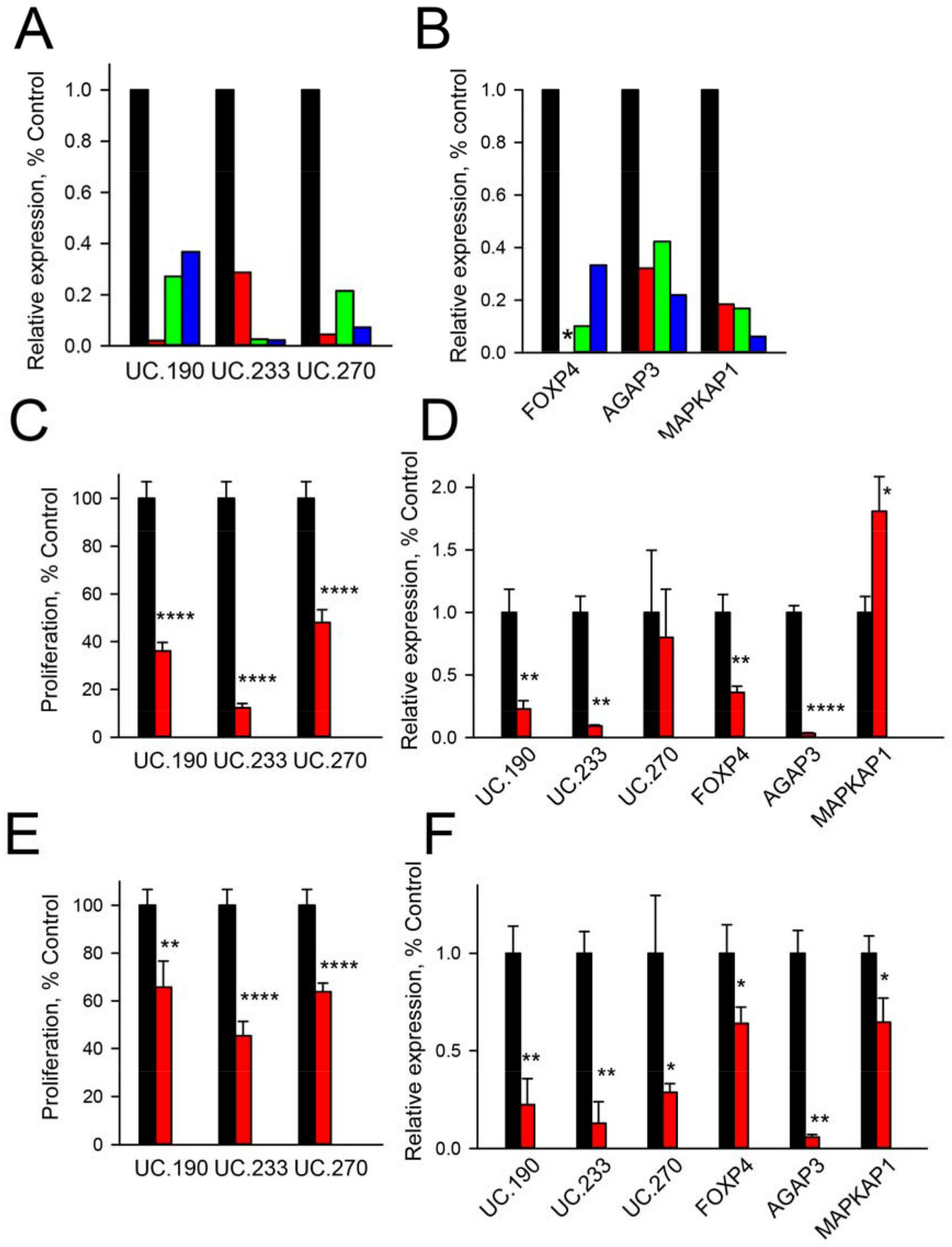

Supplementary Figure S3: Validation of T-UCR and host gene expression following siRNA or gapmer knockdown in pancreatic cancer cell lines. MIA PaCa-2 cells were exposed to 40 nM of control siRNA (black bars) or siRNA to UC.190, UC.233 or UC.270 for 96 h. Three different siRNAs-siRNA_1 (red), siRNA_2 (green) and siRNA_3 (blue) were evaluated for each T-UCR. (A) The expression of each **A.** T-UCR or **B.** T-UCR host gene was determined by qPCR. MIA PaCa-2 **C, D.** or PANC-1 **E, F.** cells were exposed to 100 nM of control (black bars) or gapmer (red bars) to UC.190, UC.233 or UC.270. (C, E) The effect on cell proliferation following a 96 h exposure to control of T-UCR specific gapmer. (D, F) The expression of each T-UCR or T-UCR host gene was determined by qPCR following 48 h exposure to gapmer.

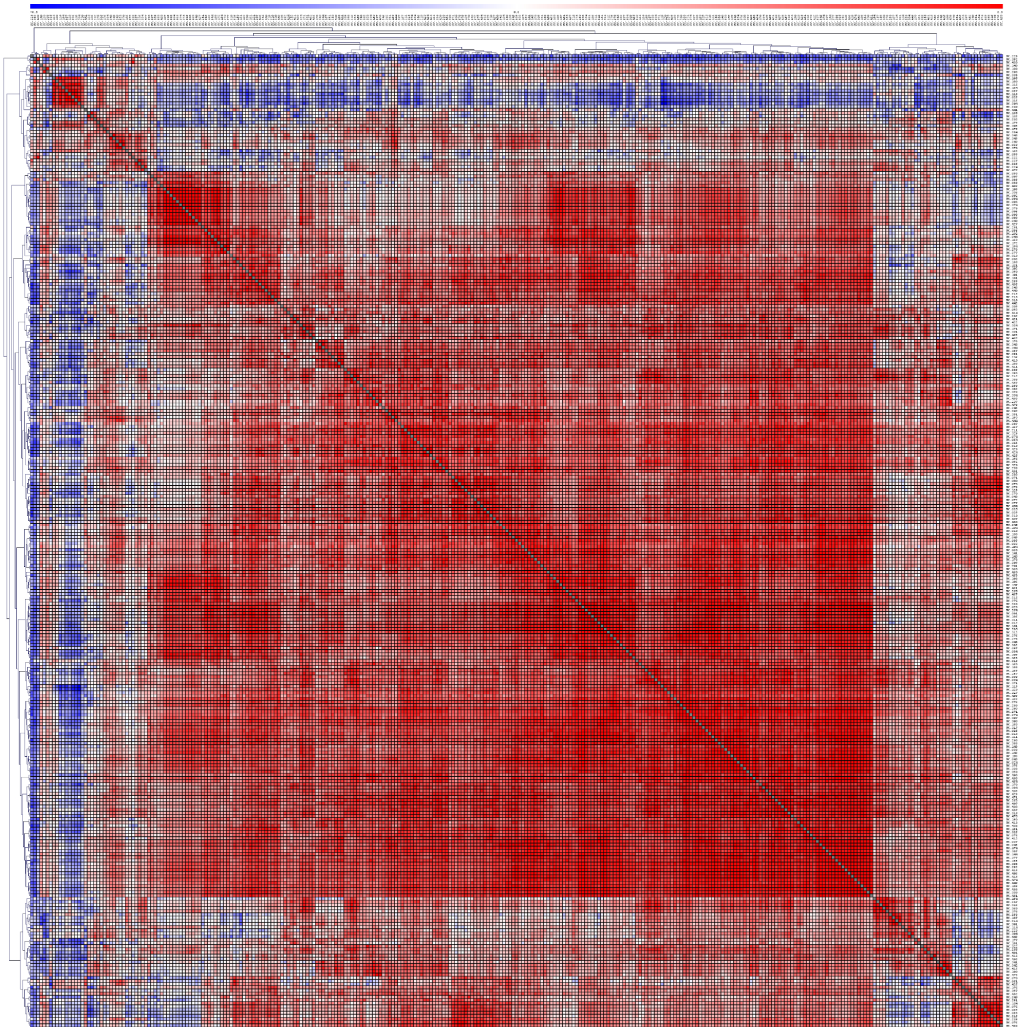

Supplementary Figure S4A: Pearson correlations for T-UCR gene expression in human tissues. Of the 481 T-UCRs profiled by qPCR in 24 specimens of PDAC, adjacent benign and normal pancreas tissues, 307 were independently expressed. The pairwise Pearson correlation coefficients for all 307 expressed TUCRs in these specimens were determined. Data are presented as heatmaps with red having the highest correlation and blue the lowest. This figure represents a high resolution image of Figure 4E.

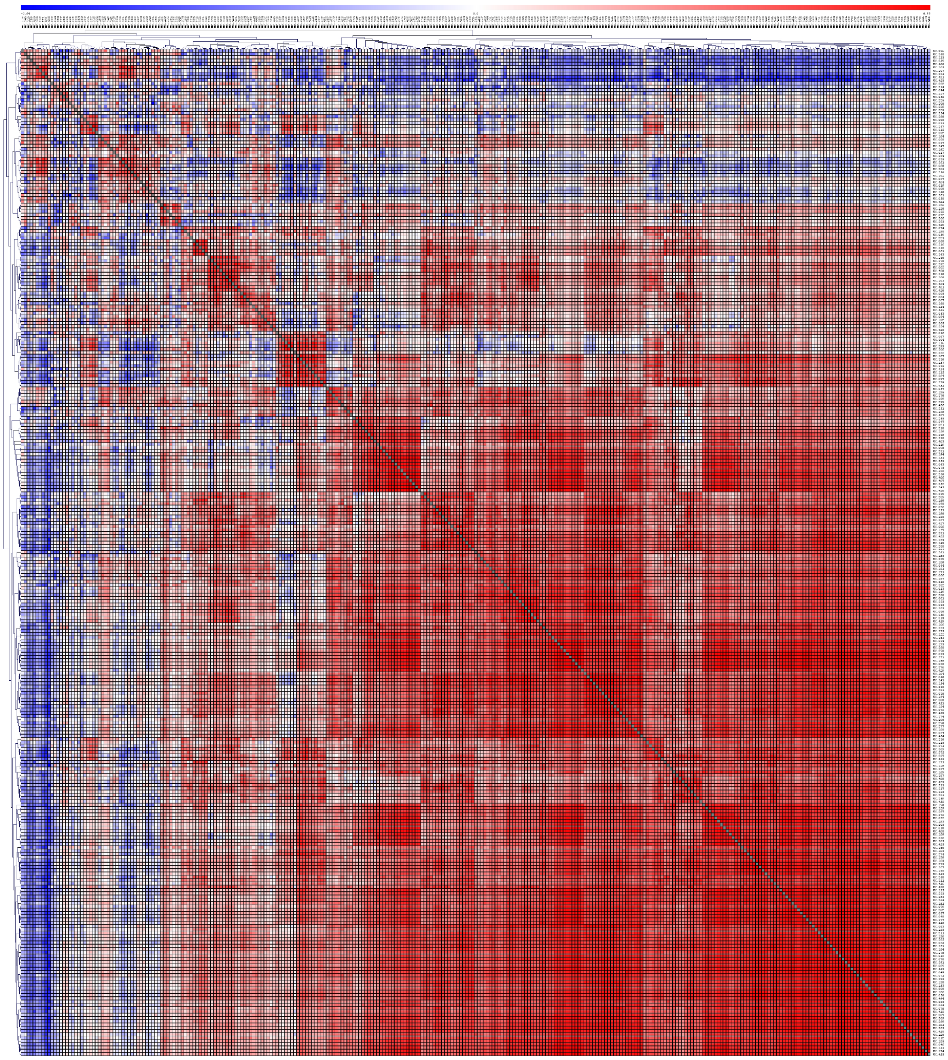

Supplementary Figure S4B: Pearson correlations for T-UCR gene expression in mouse tissues. Of the 481 T-UCRs profiled by qPCR in pancreas of young and old P48Cre/wt; KrasLSL-G12D/wt, Pdx-1-Cre; KrasLSL-G12D/wt and control mice, 328 were independently expressed. The pairwise Pearson correlation coefficients for all 328 expressed T-UCRs in these specimens were determined. Data are presented as heatmaps with red having the highest correlation and blue the lowest. This figure represents a high resolution images of Figure 4F.

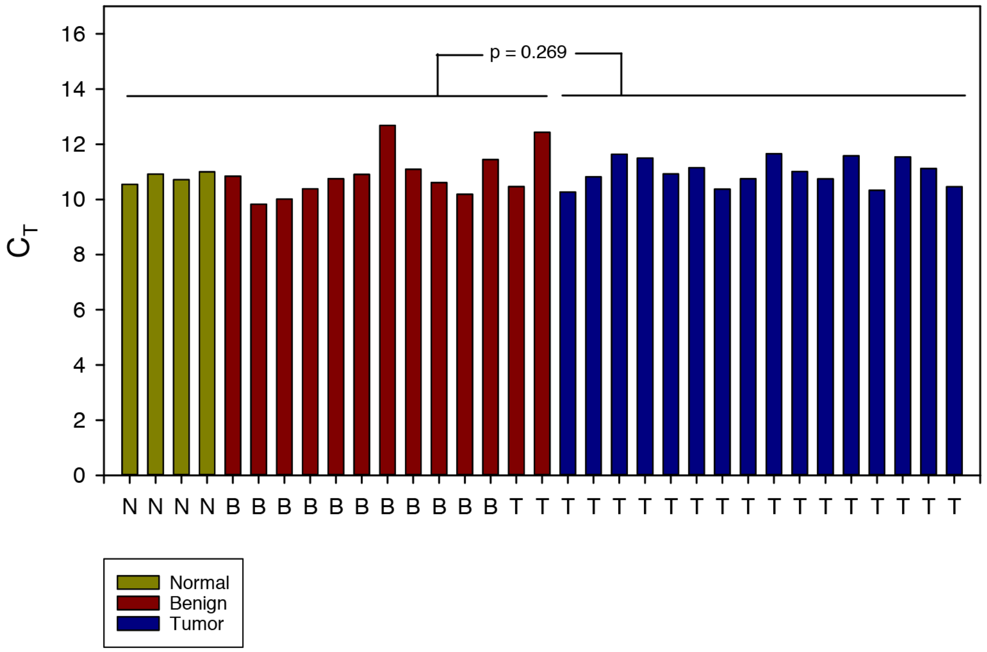

Supplementary Figure S5: 18S rRNA expression across all human tissues analyzed. The expression of 18S rRNA was examined by qPCR in specimens of PDAC (T) as well as in normal (N) and adjacent benign pancreas (B).

Supplementary Table S1: T-UCR expression (relative to 18S rRNA) in pancreas and PDAC tissues (data presented inlcude only those T-UCRs that are expressed as defined in Materials & Methods).

See Supplementary File 1

Supplementary Table S2: T-UCR expression (relative to 18S rRNA) in KRAS G12D and control mice (data presented inlcude only those T-UCRs that are expressed as defined in Materials & Methods).

See Supplementary File 2

Supplementary Table S3: T-UCR expression (relative to 18 S rRNA) in normal and PDAC cell lines (data presented inlcude only those T-UCRs that are expressed as defined in Materials & Methods).

See Supplementary File 3

Supplementary Table S4: T-UCR expression (relative to 18S rRNA) in HPDE cultured on Panc-1 extracellular matrix compared to HPDE matrix (data presented inlcude only those T-UCRs that are expressed as defined in Materials & Methods).

See Supplementary File 4

Supplementary Table S5: Fold change and p values, tumor versus normal and benign tissue, data set GSE71990.

See Supplementary File 5

Supplementary Table S6: Information on cell line authentication.

See Supplementary File 6

Supplementary Table S7: Patient data.

See Supplementary File 7

Supplementary Table S8: Age and RNA integrity number (RIN) of transgenic animals.

See Supplementary File 8

Supplementary Table S9: Primer sequences.

See Supplementary File 9

Supplementary Table S10: Validation of normalizers for qRT-PCR.

See Supplementary File 10

Supplementary Table S11: siRNA sequences.

See Supplementary File 11
